# Co-design, Co-production, and Co-evaluation Processes for a Mobile Health Check-Up Research Project in Jaipur, India: A Case Study of the Portable Health Clinic, 2016–2020

**DOI:** 10.1007/978-981-15-8632-3_3

**Published:** 2020-09-18

**Authors:** Fumihiko Yokota, Manish Biyani, Rafiqul Islam, Ashir Ahmed, Mariko Nishikitani, Kimiyo Kikuchi, Rieko Izukura, Yasunobu Nohara, Naoki Nakashima

**Affiliations:** The Institute of Decision Science for a Sustainable Society (IDS3), Kyushu University, Fukuoka, Fukuoka Japan; 2 Institute of Decision Science for Sustainable Society, Kyushu University, Fukuoka, Japan; 3 Biyani Group of Colleges, Jaipur, Rajasthan India; 4 School of Materials Science, Japan Advanced Institute of Science and Technology, Ishikawa, Japan; 5 Medical Information Center, Kyushu University Hospital, Fukuoka, Japan; 6 Graduate School of Information Science and Electrical Engineering, Kyushu University, Fukuoka, Japan

**Keywords:** Healthcare services, Disease prevention, Transdisciplinary research, Co-design, Co-implementation, Co-evaluation, India

## Abstract

This chapter summarizes the co-design, co-production, and co-evaluation processes of a mobile health check-up research project in Jaipur, India, from March 2016 to June 2020. It is the continuation of our previous paper which was published in November 2018 at *Sustainability*. The main focus of this chapter is to describe the processes of co-production, co-implementation, and co-evaluation research activities after November 2018. To accomplish this, all documents and materials related to the research processes of co-design, co-production, and co-evaluation were thoroughly reviewed, including minutes from meetings, consultations, workshops, trainings, presentation slides, pictures, and reports. After reviewing the past 4 year’s research process, the road map of a sustainable mobile health check-up project in India was proposed.

## Introduction

Future Earth research incorporates both natural and social sciences to solve global environmental issues (Lovbrand et al. 2015) and has “pioneered approaches to co-design and co-production of solutions-oriented transdisciplinary research for global sustainable development” (Future Earth 2014). However, transdisciplinary research is still hampered by a number of issues. First, the most recent reviews of transdisciplinary case studies concluded that in the co-design and co-production, processes of transdisciplinary research, methods, and concepts are still not clearly framed (Moser 2016; Brandt et al. 2013; Leemans 2016; Zscheischler et al. 2017). Second, most previous literature pertaining to transdisciplinary research only describe an early stage of “co-design” rather than actual implementation, application, and evaluation stages (Brandt et al. 2013; Page et al. 2016). Previous research includes the key components of early co-design phases such as “framing of problems” (Leemans 2016; Zscheischler et al. 2017; Adler et al. 2018), “social capital,” “partnership with mutual trust” (Mckee et al. 2015; Emmons et al. 2008; Ruddy and Rhee 2005), “scaling” (Fraser et al. 2006), “accountability” (Van Del Hel 2016; Lang et al. 2012), “ownership” (Lang et al. 2012), and “priorities and needs” (Rhodes et al. 2012). However, a few previous transdisciplinary research have reported on participatory case studies including “co-design,” “co-production,” and “co-evaluation” together in a comprehensive, bottom-up manner (Leemans 2016). Finally, fewer transdisciplinary case studies, particularly on health issues, have been conducted in low- and middle-income countries in Asia, compared to Europe and North America (Leemans 2016). Therefore, the whole processes of co-design, co-production, and co-evaluation in health-related transdisciplinary research in Asia are still vague and largely unknown.

**Fig. 3.1:**
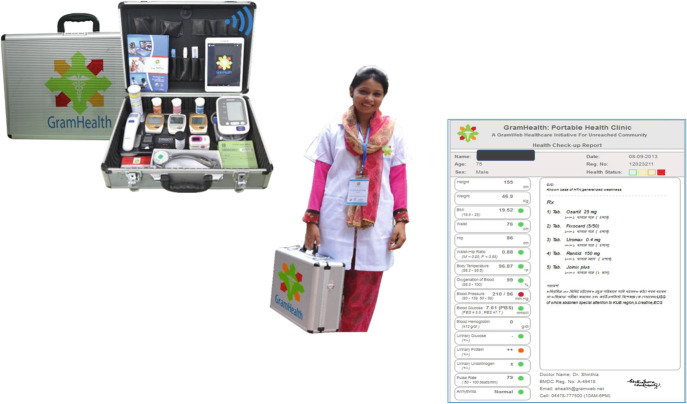
Portable health clinic box, healthcare entrepreneur, and health check-up result

To fill these knowledge gaps, a community-based mobile health check-up research project called “Portable Health Clinic (PHC)” was introduced in Jaipur, India, in March 2016 as part of a collaborative Future Earth research project among Kyushu University (KU), Biyani Group of Colleges (BGC), and Grameen Communications (GC) (Yokota et al. 2018) (Fig. 3.1). This paper is the continuation of our previous paper which was published in November 2018 at *Sustainability* and aims to update and describe the processes of this health-focused transdisciplinary research that are still taking place as of June 2020. Based on the reviews of the past 4 year’s research process, the road map of a sustainable business model in the mobile health check-up project was proposed. As an initial step, we hope to contribute to developing a methodological and conceptual framework for health-focused transdisciplinary research, as well as a new model for collaborative processes in the Asian context.

## Portable Health Clinic (PHC) Research Project

A remote healthcare system called PHC was originally developed and implemented in Bangladesh in 2010 under a research collaboration agreement between GC and KU in 2007. GC is one of the Grameen family organizations established by Dr. Muhammad Yunus, the Nobel Peace Prize-winning founder of Grameen Bank. It is a non-profit information technology company in Bangladesh that provides software products and services, Internet services, hardware and networking services, and IT education (Wikipedia 2018). PHC is an e-health service system that includes a set of medical sensor devices in a briefcase allowing mobile health check-ups and telemedicine services in remote rural areas using Skype (Nohara et al. 2015; Ahmed et al. 2014; Nakashima et al. 2013) (Fig. 3.1). After Kyushu University Hospital joined the PHC project in 2012, PHC focused on the prevention and management of noncommunicable diseases (NCDs) such as diabetes, hypertension, and obesity. At the beginning of 2019, PHC services had been used by more than 42,000 people at 32 locations in Bangladesh (Grameen Communications 2020).

### Data Sources

Data sources for this paper were all the research reports, activity logbooks, presentation slides, and the research plan/protocol including research timelines, activity schedules, and budgets. These documents produced during the period between March 2016 and June 2020 were thoroughly reviewed.

#### Co-design and Co-production Processes Among KU, GC, and BGC in Phase 1 (Photo 3.1)

**Fig. 3.2:**
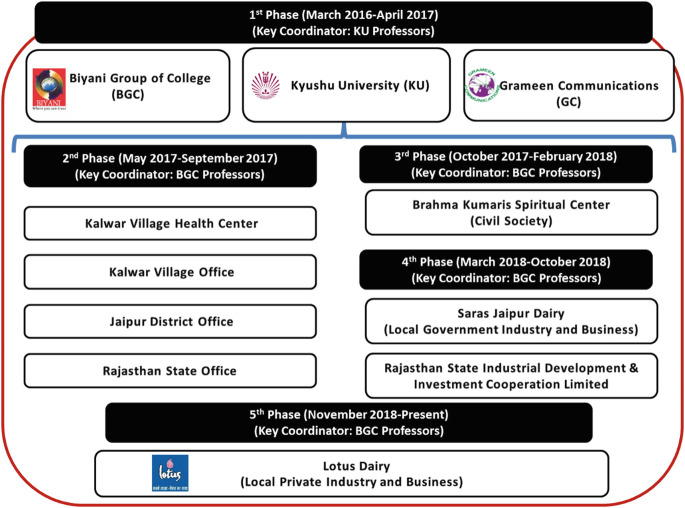
Stakeholders and key coordinators for the PHC research project in Jaipur in five phases (March 2016 to September 2019)

In India, the PHC research project started in March 2016 as a community-based health check-up service as a collaboration between KU, GC, and BGC as part of the Future Earth Research Project funded for the Institute of Decision Science for Sustainable Society, KU (Fig. 3.2). For more detailed information on the processes in phase 1, please refer to Yokota et al. (2018).

**Photo 3.1:**
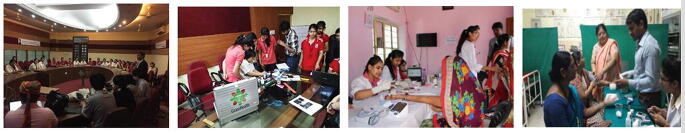
Initial meeting, staff training, and pilot implementation jointly conducted by KU, GC, and BGC in phase 1 (March 2016 to April 2017)

#### Co-design Process with Local Government Agencies in Phase 2 (Photo 3.2)

**Map 3.1:**
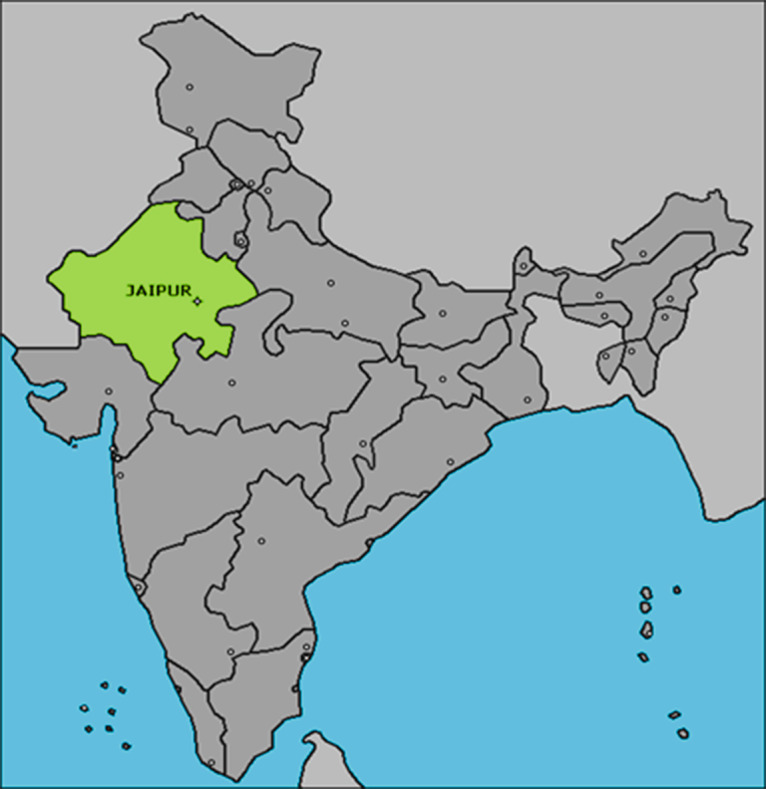
Location of Rajasthan state and Jaipur city

After going through the pilot phase 1, KU and BGC realized that permission and agreement from local government agencies were necessary for officially implementing the PHC research project in Rajasthan (Map 3.1). In May 2017, the research team visited the following agencies: Rajasthan state and Jaipur district department of medical, health, and family welfare; Kalwar village office; and Kalwar health center (Fig. 3.2). For more detailed information on the processes in phase 2, please refer to Yokota et al. (2018).

**Photo 3.2:**
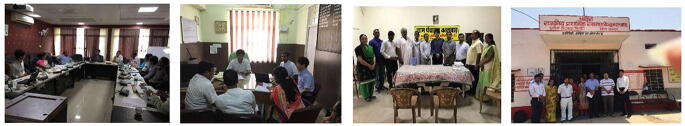
Courtesy visits including meetings and discussions held among government stakeholders (from left: Rajasthan state, Jaipur district, Kalwar village government office, and Kalwar Health Center) in phase 2 (May 2017 to September 2017)

#### Co-design, Co-production, and Co-evaluation Processes with a Civil Society Organization in Phase 3 (Photo 3.3)

In phase 3, starting from October 2017, KU and BGC had signed the official academic collaboration agreement. Based on the revised research plan and protocol, we decided to investigate the health needs, priorities, and longitudinal effects of our PHC health check-up services among different population cohort groups in various Jaipur districts. To achieve these objectives, we needed to follow up with the same individuals over time to monitor changes in their health status, behaviors, needs, and priorities. In this aspect, members of civil society organizations were much easier to follow up with than general community residents over the years. Thus, BGC first contacted the Brahma Kumaris World Spiritual University (BK) to be a stakeholder and target research population group (Fig. 3.2). For more detailed information on the processes in phase 3, please refer to Yokota et al. (2018).

**Photo 3.3:**
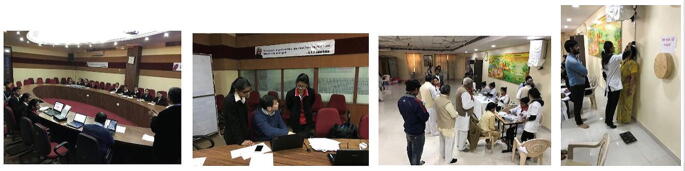
Seminars, workshop trainings, and pilot implementation jointly conducted by KU, BGC, and BK Center in phase 3 (October 2017 to February 2018)

#### Co-design, Co-production, and Co-evaluation Processes with Local Government Industry in Phase 4 (Photo 3.4)

In phase 4, starting January 2018, the core research objective was the same as in phase 3, to understand the health status, behaviors, and needs of local people as well as determining the effectiveness and acceptability of our project in a Jaipur district. However, in phase 4, the research target population was industry employees rather than general community residents or civil society members. Our aim was to identify the high-risk population groups which are most in need of health check-up services for NCD prevention. Thus, the study in phase 4 enables the comparison of health needs, health status, and health behaviors among civil society organization members, industry employees, and the general community members in Rajasthan. For more detailed information on the processes in phase 4, please refer to Yokota et al. (2018).

**Photo 3.4:**
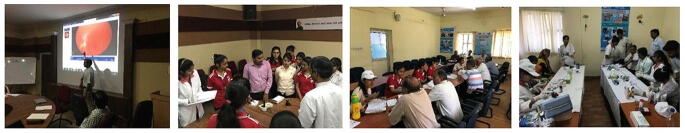
Seminars, workshop trainings, pilot implementation jointly conducted by KU and BGC, at Saras Dairy and BGC sites in phase 4 (March 2018 to October 2018)

#### Co-design, Co-production, and Co-evaluation Processes with Local Private Industry in Phase 5 (Photo 3.5)

In phase 4, PHC services could not continue as a sustainable business model nor health insurance scheme in a sustainable manner. From the previous phrases, we learned that there are needs for eye check-up and traditional Indian doctors (Ayurveda) among factory employees in Jaipur district, India. In addition, factory employees need more detailed blood tests including HbA1c for diabetes and HDL/LDL cholesterol. Therefore, the Indian PHC services were customized according to specific population’s needs. We also learned that it was very difficult to implement long-term sustainable health check-up services for government factory employees due to the following reasons: (1) government system is very slow and difficult such that it has many approval steps and paper works to proceed a new health check-up scheme, (2) government factory managers (leaders) do not want to have additional tasks or extra works to start new sustainable health check-up system, and (3) government leaders request many things but not take initiatives to start a new sustainable health check-up system. In phase 5, therefore, we changed our collaborative partners from government companies to private companies because private companies do not have such obstacles (1)–(3) that government companies have. From September to October 2018, we have searched for any potential private companies who are interested in implementing health check-up services for their employees in a long term as a health insurance scheme or social business. From BGC’s principle’s network, we found that Lotus Dairy (LD) Milk Company is interested in the PHC health check-up services for their employees and they were willing to provide their own factory’s facility room as a PHC clinic and also willing to pay for a cost for medicines. KU, GC, BGC, and LD had several meetings to discuss “how can we continue to implement PHC health check-up services for LD’s employees even after research funds finish?”. Based on the discussions, all four (KU, GC, BGC, and LD) could have an official agreement on implementing sustainable PHC health check-up services for LD’s factory employees in Jaipur district, India (Fig. 3.2). However, due to the closure of KU, Institute of Decision Science for Sustainable Society, this agreement had to be ended by the end of March 2020. Although the official joint research agreement was ended in March 2020, KU, BGC, GC, and LD tried to continue the project by developing the cost-sharing scheme as below:

- LD is to provide a cost for their factory clinic, electricity, water, and other clinic maintenance cost as well as the cost for medical drugs.
- BGC is to provide a cost for human resources (coordinator, Ayurveda doctor, health workers, IT technicians).
- GC is to provide technical assistance on data management and software for PHC system.
- KU is to provide scientific knowledge and evidence on the effects and impacts of health check-up and tele-consultation services (data collection, data analysis, publish academic papers, etc.) and provide consumables for health check-up for the first 1 or 2 years.

**Photo 3.5:**
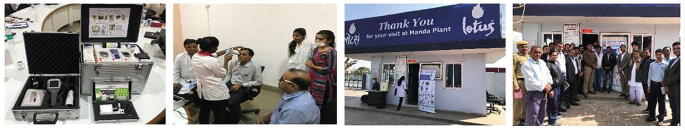
Newly customized PHC provided for factory employees by KU, GC, and BGC, at Lotus Dairy and Lotus Manda factory clinic in phase 5 (November 2018 to Present)

**Fig. 3.3:**
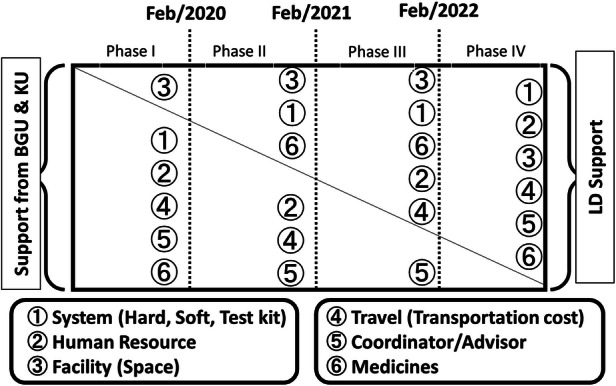
Road map of the proposed LD company’s health insurance business model of PHC project, India

In addition, KU and BGC developed a road map of the proposed business model for LD to be self-sustained in this PHC project (Fig. 3.3). In February 2020, KU, BGC, and LD had a face-to-face meeting at the LD headquarter office at Jaipur, India, to discuss the possibility of continuing the PHD project as the proposed business model. LD made two requests:

1. More detailed evaluation results collected from company employees who received the PHC services including their satisfaction levels
2. More robust supports and promotion from local government agencies such as the Ministry of Health, Medicine, and Family Welfare in India

KU and BGC agreed to have the evaluation interview survey for those who received PHC services and request LD for jointly implementing the second time PHC services and evaluation surveys targeting those who received PHC services once before. Unfortunately, due to the COVID-19 situations in India and Japan, our joint PHC project has been pending as of June 2020.

#### Summary of Co-design and Co-production Processes in All Five Phases

**Fig. 3.4:**
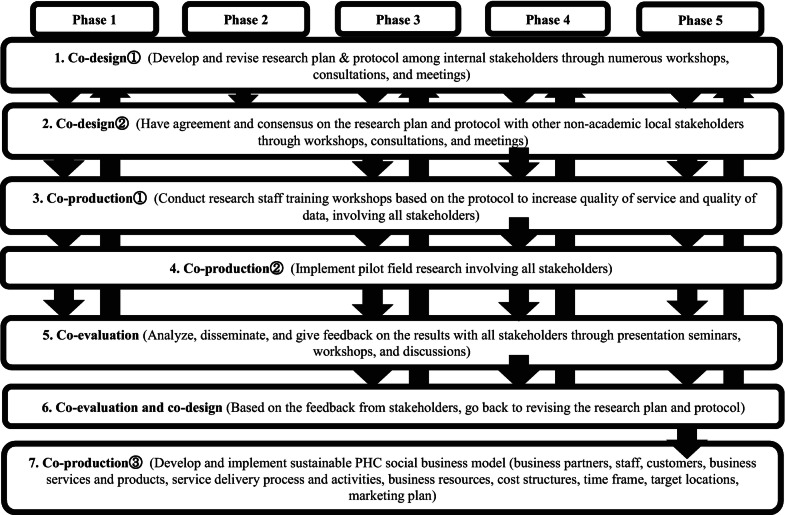
Co-design, co-production, and co-evaluation process of India PHC project

In all phases that we described in this section, we utilized the following co-design, co-production, and co-evaluation process steps (Fig. 3.4): (1) jointly develop and revise a research plan and protocol which includes research objectives, methods, timelines, activity schedules, and budgets; (2) reach collaborative agreement and consensus on the research plan and protocol with both academic and nonacademic stakeholders through meetings, consultations, and workshops; (3) conduct local research staff training workshops based on the protocol in order to improve the quality of services and quality of data jointly with stakeholders; (4) implement the pilot PHC health check-up research involving all stakeholders; (5) analyze, disseminate, and provide feedback on the results with all stakeholders through presentations, seminars, workshops, and conferences; (6) revise the research plan and protocol based on feedback such as local needs, priorities, and requests from stakeholders; and (7) develop and implement sustainable PHC social business model (business partners, staff, customers, business services and products, service delivery process and activities, business resources, cost structures, time frame, target locations, marketing plan). We should have engaged with all of our government, industry, and community stakeholders at an earlier stage of the co-design process, so that we did not need to go and back again between co-design and co-production. Van der Hel et al. (2016) indicated that engaging more stakeholders throughout the research process will increase legitimacy and reduce skepticism. Some key factors, such as effective local coordinators, personality types of stakeholder leaders, and continuous involvement and engagement with stakeholders, particularly both government and nongovernment stakeholders helped mitigate such difficult situations in our research in India.
